# Engaging patients and primary care providers in the design of novel opinion leader based interventions for acute asthma in the emergency department: a mixed methods study

**DOI:** 10.1186/s12913-018-3587-7

**Published:** 2018-10-19

**Authors:** Cristina Villa-Roel, Maria Ospina, Sumit R Majumdar, Stephanie Couperthwaite, Erin Rawe, Taylor Nikel, Brian H Rowe

**Affiliations:** 1 Department of Emergency Medicine, University of Alberta, 7-30 University Terrace, 8303 - 112 Street, Edmonton, AB T6G 2T4 Canada; 2 Department of Obstetrics & Gynecology, University of Alberta, 7-30 University Terrace, 8303 - 112 Street, Edmonton, AB T6G 2T4 Canada; 3 Departments of Medicine, University of Alberta, 7-30 University Terrace, 8303 - 112 Street, Edmonton, AB T6G 2T4 Canada; 4 School of Public Health, University of Alberta, 7-30 University Terrace, 8303 - 112 Street, Edmonton, AB T6G 2T4 Canada

**Keywords:** Asthma, Exacerbations, Education, Knowledge translation

## Abstract

**Background:**

Multifaceted interventions driven by the needs of patients and providers can help move evidence into practice more rapidly. This study engaged both patients and primary care providers (PCPs) to help design novel opinion leader (OL)-based interventions for patients with acute asthma seen in emergency departments (EDs).

**Methods:**

A mixed methods design was employed. In phase I, we invited convenience samples of patients with asthma presenting to the ED and PCPs to participate in a survey. Perceptions with respect to: a) an ideal OL-profile for asthma guidance; and b) content, style and delivery methods of OL-based interventions in acute asthma directed from the ED were collected. In phase II, we conducted focus groups to further explore preferences and expectations for such interventions with attention to barriers and facilitators for implementation.

**Results:**

Overall, 54 patients completed the survey; 39% preferred receiving guidance from a respirologist, 44% during their ED visit and 56% through individual discussions. In addition, 55% expressed interest in having PCP follow-up within a week of ED discharge. A respirologist was identified as the ideal OL-profile by 59% of the 39 responding PCPs. All expressed interest in receiving notification of their patients’ ED presentation, most within a week and including diagnosis and ED/post ED-treatment. Personalized, guideline-based, recommendations were considered to be the ideal content by the majority; 39% requested this guidance through a pamphlet faxed to their offices. In the focus groups, patients and PCPs recognized the importance of health professional liaisons in transitions in care; patient anxiety and PCP time constraints were identified as potential barriers for ED-educational information uptake and proper post-ED follow-up, respectively.

**Conclusions:**

Engaging patients and PCPs yielded actionable information to tailor OL-based multifaceted interventions for acute asthma in the ED. We identified potential facilitators for the implementation of such interventions (e.g., patient interaction with alternative health care professionals who could facilitate transitions in asthma care between the ED and the primary care setting), and for the provision of post discharge self-management education (e.g., consideration of the first week of ED discharge as a practical time frame for this intervention). Prioritization of identified barriers (e.g., lack of PCP involvement) could be addressed by the identification of potential early adopters in practice environments (e.g., clinicians with special interest in asthma).

## Background

Asthma is a chronic lung disease characterized by airway inflammation and patients with this disease may experience episodes of flare-ups, often in response to a variety of triggers. Despite marked improvement in the understanding of the pathophysiology of asthma [1] and broad therapeutic advances, control of asthma symptoms remains elusive for many patients [2]. This loss of control results in frequent exacerbations which, when severe, may result in emergency department (ED) visits [3].

While the pharmacological ED management of acute asthma has been recognized as “evidence-based” [4], there is a need to facilitate the transitions in care between acute care settings such as the ED and community-based primary care providers (PCPs). Important knowledge and care gaps have been identified in adults presenting to EDs with asthma exacerbations [5]; some of these gaps are more common in subjects at high risk of admissions and relapses [6, 7]. In addition, research has shown that follow-up visits with a PCP after ED discharge are delayed or non-existent [8]. Recently published and widely disseminated asthma guidelines have highlighted the essential role of patient education and the establishment of post-ED care partnerships after an asthma exacerbation [9]. These strategies are designed to regain and maintain asthma control, prevent poor health outcomes and maximize patients’ quality of life; however, the evidence supporting the use of such guidance in practice is mixed [10, 11]. Consequently, this makes any plan for sustained implementation a complex and challenging endeavor [12].

Strategies to engage PCPs and encourage follow-up of patients with asthma is an important goal to achieve and should improve patient outcomes. Opinion leaders (OL) are recognized as individuals capable of using their knowledge to influence the opinions, behaviours, beliefs and attitudes of others [13, 14]. Effective, safe and responsive OL-based interventions have shown to improve professional practice and health outcomes [15]; active OL-based multifaceted interventions targeting different barriers to change have been proposed as novel strategies for knowledge transfer [16]. The involvement of OLs in the implementation of quality improvement and educational interventions in health care is not necessarily effective under all circumstances, their benefits seem to be intervention- and disease-specific [17].

Emergency department-directed OL-based interventions targeting chronic conditions, such as osteoporosis, have facilitated patient and PCPs linkages, improved PCP follow-up and resulted in large improvements in appropriate testing and treatment compared with usual care [18, 19]. While community-based influential physicians have been shown to influence the behaviour of peers, reduce their need to participate in traditional educational programs and improve care in patients with chronic obstructive disease (COPD) [20], ED-based studies comparing OL-endorsed treatment recommendations for ambulatory asthma and COPD with usual care found no increase in PCP follow-up visits at 30 days or reduction of relapses at 90 days [21].

To date, the effectiveness of OL-based multifaceted interventions facilitating the transitions in care between the ED and the primary care setting to improve health outcomes after asthma exacerbations has not been established. In addition, the specific value of letting patients and PCPs’ perceptions and expectations influence the content of these interventions has been infrequently studied [22, 23].

## Methods

### Aim

The aim of the current study was to seek input from patients and PCPs on the design of novel OL-based multifaceted interventions for acute asthma.

### Study design

A sequential explanatory mixed methods design was employed, which involved quantitative (survey) and qualitative (focus groups) data collection. The structure of the mixed-methods approach is QUAN → qual, in which quantitative methods precede qualitative and the quantitative methods are dominant [24]. This sequential approach serves the function of convergence and complementarity to seek elaboration and clarification of survey results. The study was approved by the University of Alberta Health Research Ethics Board (Pro00023191; Tailoring Educational Interventions in Acute Asthma). Written informed consent was waived; patients and PCPs’ voluntary responses/participation reflected their consent to take part in the study.

### Quantitative methods (surveys)

Over a four-month period, a consecutive sample of at least 50 patients with physician-diagnosed asthma who had ever presented to the ED for acute asthma were invited by trained research assistants to complete a self-administered survey, regardless of their reason for ED presentation to the University of Alberta Hospital. Information on demographics, primary care support and perceptions of an ideal OL-profile for the provision of guidance in ambulatory asthma care were collected; preferences regarding the content, style and delivery methods of OL-based interventions in acute asthma directed from EDs were also gathered. Options for an OL included as a respiratory medicine specialist (“respirologist”), an internal medicine specialist, or a family physician with or without special interest in asthma. The survey took approximately 10 min to complete.

During the same period of time, a random sample of 50 PCPs (family physicians from the Edmonton area) was chosen from the College of Physicians and Surgeons of Alberta website (www.cpsa.ab.ca) and each was invited to participate in an electronic survey. Due to a *null* response to the electronic survey, surveys were subsequently faxed to a second random sample of 150 PCPs chosen from the same website. Due to a low response to this second attempt, surveys were distributed during an academic event involving family physicians from the Edmonton area. Apart from the same information collected in the patient survey, training designation and years of clinical experience were documented.

At the end of both surveys, patients and PCPs were invited to participate in separate focus groups to discuss their responses, to further explore their preferences and expectations regarding the delivery of ED-directed interventions for asthma care involving OLs, and to debate their potential impact on clinical practice.

### Qualitative methods (focus groups)

Focus group questions were developed following a semi-structured format with open-ended questions [25]. Introductory questions were developed to build rapport and encourage an open discussion among participants. After introductions, survey results were presented and focus group moderators asked a series of probing questions about participants’ preferences on the delivery of ED-directed educational interventions for asthma care involving OLs; the ideal content, style and delivery methods to improve patient-PCP linkages were also sought. Potential barriers for intervention implementation and knowledge uptake were also explored.

Two focus group discussions (2 h each) were conducted with the same instructions and questions. One researcher with experience in qualitative research moderated them with the aid of two clinician-researchers. Focus groups were audio recorded; however, two process facilitators also completed field notes that documented the main themes of the session and any observations pertinent to the study aims. Focus group discussions and notes were transcribed verbatim.

### Data analysis

The results of the surveys were analyzed and summarized using descriptive statistics: proportions for categorical variables and medians with percentiles and interquartile range (IQR: P_25_, P_75_) for continuous variables (due to a non-normal distribution of the data). Data were analyzed using Stata Statistical Software® Release 13.0 (College Station, TX, Stata Corporation).

A conventional approach to content analysis was used to create coding categories and identify themes and patterns derived directly from the data obtained from the lived experience at the focus groups [26, 27]. Patient and PCPs’ responses (including the identification of potential barriers and facilitators for potential implementation) were interpreted from the content of text data and not from a pre-existing theoretical framework. Data transcripts were condensed into text segments that were coded based upon emergent themes that were continually refined and compared to each other. Finally, categories were aggregated into broader themes related to participants’ preferences, expectations and views on barriers and facilitators for the delivery of ED-based education interventions for asthma. Excerpts from participants’ narratives were used to illustrate the main themes derived from focus group discussions.

## Results

**Fig. 1:**
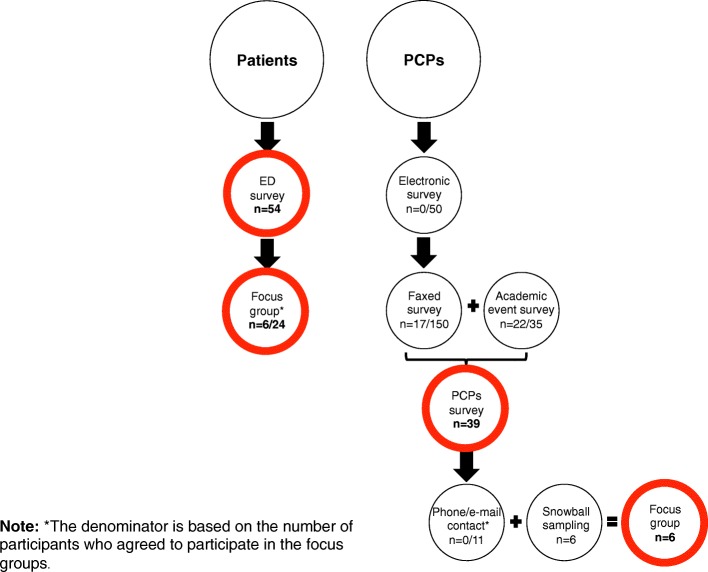
Study recruitment strategies. ED = Emergency department; PCPs = Primary care providers

Figure 1 summarizes the study recruitment strategies and the response/participation for the surveys and the focus groups, respectively.

### Patient survey results

A total of 54 patients with asthma completed the survey; their median age was 44 (IQR: 27, 58) years and 55% were female. Overall, 65% of patients reported a family physician frequently managed their asthma; 39% preferred to receive guidance regarding their asthma exacerbation from a respirologist, 44% during their ED visit and 56% through one-on-one discussions. In addition, 55% expressed interest in having PCP follow-up within a week of being discharged from the ED; however, the difficulty obtaining a follow-up visit was reported as moderate on a 1–7 Likert scale ranging from very difficult (1) to very easy (7); the median was 4 (IQR: 3, 6).

### Primary care provider survey results

The response rates to the PCP faxed-surveys and to the surveys handed-out in an academic event were 11% (*n* = 17/150) and 63% (*n* = 22/35), respectively. A total of 39 PCPs completed the survey; 39% of them were in the 46–55 years age category and 72% were female. A respirologist was identified as the ideal OL-profile for the provision of guidance in ambulatory asthma by 59% of the respondents. All PCPs expressed interest in receiving notification of their patients’ ED acute asthma presentation; 62% considered personalized, guideline-based, recommendations to be the ideal content of an educational intervention directed from the ED and 39% were inclined to receive this guidance through an educational pamphlet faxed to their offices. Finally, 54% preferred this notification within a week of ED discharge including details on: ED treatment (95%), final diagnosis (92%), and post-ED treatment (87%).

### Focus group results

Findings include a description of participants’ characteristics, preferences, expectations and views on barriers and facilitators for the delivery of OL-based educational interventions for asthma directed from the ED. Table 1 summarizes patients and PCPs’ illustrative statements for the main themes that emerged from these activities.

**Table 1:** Patients and primary care providers’ statements for the main themes that emerged from the focus groups

Participants	Main themes	Statements
Patients with asthma	Preference for specialized knowledge in the delivery of ED asthma education	“They know what to prescribe to you. They are a lot more specialized or they will have more idea what to give you for what your symptoms are”.
	Anxiety as barrier for information uptake during the ED visit	“You are scared, you’re terrified; you are focused on your breathing. Honestly I thought I was dying”.
	Role, content and provider of “teachable moments” in the ED	“I would sit with a nurse or whoever and talk while I am in the actual emergency area. But I don’t think I am taking information in. You could talk to me until I’m blue in the face but if I’m not well and having an asthmatic attack, I’m telling you I’m not taking the information in because I am not thinking”.
	Transitions in care from emergency to the primary care settings	“Sometimes you go to see your family doctor and although they are trying to give you the best care that they can, they are so overwhelmed a lot of times with their practice that they don’t always have full time for you, whereas if you go to see your lung specialist, that’s basically all they are there for your problem. Your family doctor can’t do the tests that the asthma doctors do”.
Primary care providers	Notification and timing of follow-up after ED discharge	“Why can’t [I] get this a day after?; everybody wants notification that his/her patient has been in Emerg. Realistically within a day is not going to happen, but it has to be as soon as practical. If they don’t recover from the episode, I want to know that day. If they weren’t given prednisone or ICS, it would be good to know. Three weeks later, they’re going to be in real trouble”.
	Content of ED discharge letters and education	“Diagnosis is the key part here. The diagnosis of asthma would make me act, it’s not about doubting the diagnosis, it’s used like a red flag/alert (it’s nice to be able to read the diagnosis/be given a diagnosis, then you know what to do.”
	Role of OLs for ambulatory asthma care and education	“Family physician perspective is better, more relatable, getting taught by people that know your experience, less of a top down approach”.
	Time constraints for post-ED follow-up and education	“Physicians often can’t spend hours with patients; asthma educators can review environmental changes, be more didactic; they can show pictures and graphs.”

*ED* Emergency department

### Patients

From 24 patients with asthma who completed the survey and agreed to participate in the focus group, six attended. Their median age was 55 years (IQR: 52, 58) and six were females. All but one patient had a PCP (family doctor) and reported current regular use of medication to control asthma. Analysis revealed four main themes that emerged from the focus group discussions:

- Theme 1: Preference for specialized knowledge

Patients recognized the benefits of asthma education uptake while in the ED and expressed preference for specialized education (from a respirologist) regarding the asthma episode that brought them to the ED. While great value was given to the specialized knowledge of respirologists (e.g., about the role of different medication options) patients recognized these physicians may not be available for the provision of post discharge self-management education. Other health care providers working in the ED (e.g., nurses, respiratory therapists or pharmacists) were identified as alternative clinicians who could address these topics (Table 1).

- Theme 2: Anxiety as a barrier for information uptake during the ED visit

One of the most profound themes was the role of anxiety as a barrier for asthma education in the ED. Patients reported that asthma exacerbation episodes typically trigger high levels of anxiety. The predominant message was that anxiety acted as a potential deterrent to knowledge uptake as it adversely interfered with complex cognitive processing of information (Table 1).

- Theme 3: Role, content and provider of “teachable moments” in the ED

Participants agreed that the ED offers a short window of time to receive education about asthma; however, they considered the opportunity and content of “teachable moments” may vary according to symptom severity and anxiety levels during the ED stay. Although receptive, participants expressed concerns about information overload that could prevent them from accurately remembering concepts after discharge. They mentioned that information provided in the ED sometimes is not clear and leaves them confused and with several doubts about future steps. Patients recognized the uptake of fragmented information from their interaction with several health providers during their ED stay (e.g., nurse, respiratory therapist, pharmacist, ED physician). For example, some participants acknowledged the importance of receiving information from a pharmacist regarding the appropriate use of inhalers prior to discharge; however, discussions about comprehensive chronic self-management were preferred to occur outside the ED (Table 1).

- Theme 4: Importance of transitions in care from emergency to the primary care settings

Participants acknowledged the importance of ongoing education after ED discharge to support their self-management skills. There was almost unanimous feeling that disconnection between the ED, PCPs and respirologists’ recommendations are an important barrier for a successful continuum of care.

They emphasized the importance of timely one-on-one, follow-up after discharge. There were discussions about whether follow-up should occur with their PCP (family doctor) or with a specialist. Patients indicated that a long-term relationship with a family physician facilitates follow-up with this care provider; however, patients also felt the lack of specialized training in respiratory medicine would limit their ability to order and conduct specialized tests for monitoring their condition (Table 1).

### Primary care providers

A total of 11 PCPs who completed the survey and initially indicated their interest in participating in the focus group were contacted by telephone and electronic mail. None of them accepted the invitation for the focus groups and therefore, a snowball sampling strategy was used to recruit additional research participants. Six PCPs (3 males and 3 females; all family physicians with median year of graduation: 1994 (IQR: 1989, 2004) took part in the focus group discussion. Four main themes emerged from the focus group discussions with the PCPs:

- Theme 1: Notification and timing of follow-up after ED discharge

Participants stressed the importance of having a prompt notification and follow-up with their patients after they are discharged from the ED (e.g., within 1 day to 1 week after ED discharge) and while they are still on the medication prescribed during the ED visit (Table 1).

- Theme 2: Content of ED discharge letters and education

Most participants expressed preference for receiving personalized educational information instead of general asthma information or educational pamphlets faxed to their offices. There was consensus about the importance of receiving discharge letters explicitly indicating the final ED diagnosis and discharge medications. They expressed the content of the letter would be useful to determine how soon the follow-up appointment should take place (Table 1).

- Theme 3: Opinion leaders for ambulatory asthma care and education

In contrast to PCP survey respondents, participants in the focus group expressed that family physicians would be the best OL-profile for the provision of guidance on ambulatory asthma care and education. Participants acknowledged the value of respirologists as OLs though, particularly in those patients not having family physicians or for practitioners in the “late majority” category of innovation uptake. They also perceived respirologists might be able to offer advice about patients not responding to traditional management strategies. Participants felt that family physicians had a more relatable perspective and that because many of the challenges of ambulatory asthma care are not related to treatment choices but to socio-economic issues, a family physician would be more equipped to act as an OL for education on ambulatory asthma care (Table 1).

- Theme 4: Time constraints for proper post –ED follow-up and education

There was general consensus that time constraints are an important barrier for asthma education in ambulatory care settings. Participants expressed that other health providers such as asthma educators and chronic care managers could help overcoming the challenges and gaps in the delivery of asthma education in the ambulatory care setting (Table 1).

## Discussion

Input from patients and PCPs regarding the content, style and delivery methods of OL-based interventions in acute asthma directed from the ED provided valuable information for the design and implementation of novel multifaceted interventions (NCT01079000). The identification of respirologists as the ideal OL-profile for the provision of asthma guidance; of the first week after ED discharge as a practical window for education on self-management; and of one-on-one vs. personalized written materials faxed to offices as desirable delivery methods for patients and PCPs, respectively, are concrete examples of intervention components that could facilitate the design and implementation of OL-based interventions in acute asthma directed from the ED. The focus groups allowed the reconciliation of the discrepancies with the survey responses through the identification of the main driver behind the OL-profile selection: the preference for specialized knowledge. They also facilitated the identification of potential strategies to overcome barriers to the adoption of such interventions in practice environments (e.g., limited availability of respirologists for the provision of post discharge self-management education).

The fact that respirologists were nominated by most of the survey respondents as the ideal clinician profile to guide education after an asthma attack reflect their earned professional leadership role, trust and respect among individuals with different technical competences and status in the health system [17]. Respirologists’ knowledge, ability to order/conduct specialized tests and ability to manage patients with complex respiratory conditions were their most valued assets. While participants in the PCPs focus group expressed the belief that family physicians would be the best OLs for ambulatory asthma care and education, the value of respirologists as OLs was still acknowledged. Both patients and physicians, however, recognized the limited availability of respirologists for the provision of post discharge self-management education and discussed the potential benefit derived from empowering other health care providers working in or outside the ED to assume that role. The assistance that trained asthma educators, nurses, respiratory therapists and pharmacists can provide in transitions in asthma care between the ED and the primary care setting has been previously described [28, 29]. Finally, time constraints is another important barrier that could limit effective post-ED interaction with family physicians.

The first week of ED discharge was identified as a practical time frame for the provision of asthma education by patients and PCPs. While there is no clear evidence regarding the most effective time for the provision of asthma follow-up and education, their preference would be aligned with the current guideline recommendations for PCP contact/follow-up [30]. Additional initiatives referred to by PCP-survey respondents such as faxing them a copy of the patients’ ED chart and a personalized-letter including details on their patients’ final diagnosis, ED and post ED-treatment could also be considered in future studies [22]. These efforts have the potential to influence physicians’ behaviors (e.g., initiating contact with their patients, adjusting medication, making referrals, etc.); however, they are not part of “regular practice” in Canada and should only be recommended for implementation elsewhere after their cost-effectiveness is assessed using rigorous research methods [31]. While the health care system where the study was conducted did not employ secure hospital/ED to PCP electronic or smart-phone communication strategies; these are tools that could facilitate the adoption of such interventions in certain contexts [32].

Given the weak evidence of benefit derived from asthma education provided in the ED [33, 34], its not surprising that controversy exists regarding the superiority of this compared to other settings [4, 35]. In a chaotic environment like the ED, time for the delivery of anything but brief educational interventions directly related to the discharge and follow-up of a condition, may be difficult. Interestingly, patients identified anxiety as a potential barrier for the delivery of educational interventions in the ED. Targeting the increasing comorbidity of anxiety disorders in patients with asthma [36], which are usually triggered by episodes of loss of asthma control, could be a key and rarely-explored element to address in future studies evaluating factors influencing knowledge uptake in acute settings. Finally, time constraints were identified as a potential barrier for the provision of asthma education in ambulatory care settings. Asthma is a complex chronic disease with considerable knowledge and care gaps among those afflicted by it, and post-ED follow-up may be better left to those with specific training in the area and time to provide appropriate guidance.

### Limitations

The sequential explanatory mixed methods used in this study constitutes the main strength of our initiative to consider the perceptions of patients and PCPs in the design of ED-directed OL-based educational interventions in acute asthma. Nonetheless, our findings may not represent the perception of all asthmatics in Canada (or elsewhere) nor be generalizable to those presenting to all EDs. Efforts were made by the research staff to reach non-selective samples; however, difficulty accessing and receiving responses from PCPs, either for the survey or the in-person focus group, led to a potentially biased sample of highly engaged clinicians. The response issues presented here are consistent with previous experiences [37] and represent an important barrier for the design of individual-level interventions aiming change in healthcare professional behaviours [38]. In addition, the patient sample may have been biased as well, as the respondents were older and more often female than the samples engaged in ED-based asthma trials. While alternative practitioners (e.g., nurse practitioners) are generally not available to patients in this community and the potential role of non-traditional practitioners (e.g., acupuncturists, physiotherapists, holistic practitioners) was not explored for the same reasons, their inclusion in other settings may be quite reasonable. The sample sizes were small and data saturation was not formally assessed [39]; however, consistency in the qualitative responses was observed. Finally, this was our first attempt to engage patients and potential end-users (PCPs) in the design of OL-based interventions for acute asthma in the ED. The difficulties faced bringing together 4–10 patients and PCPs into one setting for the focus groups support the consideration of other qualitative methods (e.g., individual interviews) in future studies [40].

## Conclusions

Messages and recommendations arising from patients and PCPs helped tailor ED-directed OL-based multifaceted interventions in acute asthma to meet the local needs and expectations (NCT01079000). Further in-depth discussions of the survey responses helped to identify the main drivers of their preferences (e.g., professional trust for OL-profile selection), as well as potential determinants of knowledge uptake and for the implementation of our and similar interventions. Particularly, non-conventional effect-modifiers (e.g., OL profile, timing of the OL-intervention, time constraints affecting the post-ED follow-up visit and patient anxiety levels) might not have been discovered had a mixed methods approach not been employed.

## Abbreviations

COPD: Chronic obstructive disease
ED: Emergency department
IQR: Interquartile range
OL: Opinion leader
PCP: Primary care provider
